# Classification of Japanese Pepper (*Zanthoxylum piperitum* DC.) from Different Growing Regions Based on Analysis of Volatile Compounds and Sensory Evaluation

**DOI:** 10.3390/molecules27154946

**Published:** 2022-08-03

**Authors:** Kazuaki Yamasaki, Nami Fukutome, Fumiyo Hayakawa, Nobuo Ibaragi, Yukio Nagano

**Affiliations:** 1 Faculty of Health Science, Hyogo University, Kakogawa 675-0195, Japan; 2 Department of Food Sciences, Tokyo Seiei College, Tokyo 124-8530, Japan; namifukutome@gmail.com; 3 Institute of Food Research, National Agriculture and Food Research Organization, Tsukuba 305-8642, Japan; fumiyoha@affrc.go.jp; 4 Association for Conservation of Asakura Sanshō, Yabu 667-0021, Japan; rinibaragi@yahoo.co.jp; 5 Analytical Research Center for Experimental Sciences, Saga University, Saga 840-8502, Japan; nagano@cc.saga-u.ac.jp

**Keywords:** sanshō, flavor analysis, napping method

## Abstract

The Japanese pepper (*Zanthoxylum piperitum* DC.) is an attractive plant that is highly palatable and benefits human health. There are several lineages of pepper plants in Japan. However, the classification of each lineage by analyzing its volatile compounds and studies on the effects of differences in volatile compounds on human flavor perception have not been performed in detail. Herein, we conducted gas chromatography (GC) and GC/mass spectrometry (GC/MS) analysis of volatile compounds and sensory evaluation of flavor by an analytical panel using 10 commercially available dry powdered Japanese pepper products from different regions. GC and GC/MS analysis detected limonene, β-phellandrene, citronellal, and geranyl acetate as the major volatile compounds of Japanese peppers. The composition of volatile compounds showed different characteristics depending on the growing regions, and cluster analysis of composition classified the products into five groups. The sensory evaluation classified the products into four groups, and the results of both classifications were in good agreement. Our results provide an important basis for proposing cooking and utilization methods that take advantage of the unique characteristics of each lineage based on scientific evidence.

## 1. Introduction

The Japanese pepper (*Zanthoxylum piperitum* DC., “sanshō” in Japanese) belongs to the Rutaceae family (so-called citrus family), genus *Zanthoxylum*. It grows wild but is cultivated in East Asian regions, including Japan. The entire plant contains a variety of citrus-specific volatile and pungent compounds, such as sanshools [1,2]. Young leaves (buds, “kinome” in Japanese) harvested in early spring, green unripe fruits harvested in spring, and ripe red fruits harvested in fall are the most commonly eaten parts and are used in various dishes according to their respective characteristics. In Japanese cuisine (washoku), which places great importance on seasonality, this plant is one of the most important foods that symbolize the changing seasons. As Japanese cuisine spreads worldwide, the ingredients from this plant will be the focus of much attention in the future. In Japan, various parts of the Japanese pepper are used in a variety of ways, but the most common use is as a powder made by drying the peel of the ripe fruit. This dried powder is sprinkled on dishes. It is used differently from the Chinese Sichuan pepper (a relative of Japanese pepper), which is added and heated in the process of cooking.

The main volatile compounds of Japanese pepper are limonene, β-phellandrene, citronellal, and geranyl acetate [1,3,4]. Recently, the mechanism of flavor perception has been elucidated, and it has been revealed that flavor, along with taste and texture, is an important factor in determining human preferences [5]. Therefore, the composition of these volatile compounds is closely related to the palatability of Japanese peppers.

In recent years, the beneficial effects of volatile compounds and aromatherapy on human health have attracted attention [6]. The volatile compounds in the essential oils of some plants have been used in traditional medicine for their antibacterial, antioxidant, and anti-inflammatory effects, as well as for stress reduction. The mechanism of action of these essential oils in the body and the active ingredients of their action remain unknown. However, in recent years, their mechanisms of action, such as their anxiety-reducing effects and anti-inflammatory action, have been demonstrated scientifically through metabolite analysis and molecular biological techniques using cultured cells [7,8,9].

Limonene, which is characteristic of citrus fruits and is also present in Japanese pepper, has been widely studied and reported to have various effects, including anti-inflammatory, antioxidant, antinociceptive, anticancer, antidiabetic, analgesic, antiviral, gastroprotective, as well as immune, sugar metabolism, and bowel regulatory effects [10,11,12]. In particular, its anticancer and anti-inflammatory effects have been demonstrated in human clinical trials [13,14,15]. Other secondary metabolites in Japanese pepper, such as citronellal and geranyl acetate, and products containing these volatile compounds, have also been reported to exhibit biological regulatory functions [16,17,18].

Separately, excessive salt intake is a major risk factor for hypertension and subsequent cardiovascular diseases, both of which have become a serious problem in recent years; therefore, the use of spices and herbs to reduce salt intake is a focus of attention [19]. Sanshools, the pungent components of Japanese pepper, have been reported to have a salty taste-enhancing effect [20]; therefore, Japanese pepper is also expected to be useful as a material for reducing salt intake. Thus, Japanese pepper is an attractive material that is not only palatable but can also benefit human health by preventing and improving metabolic, non-infectious, and infectious diseases.

**Figure 1:**
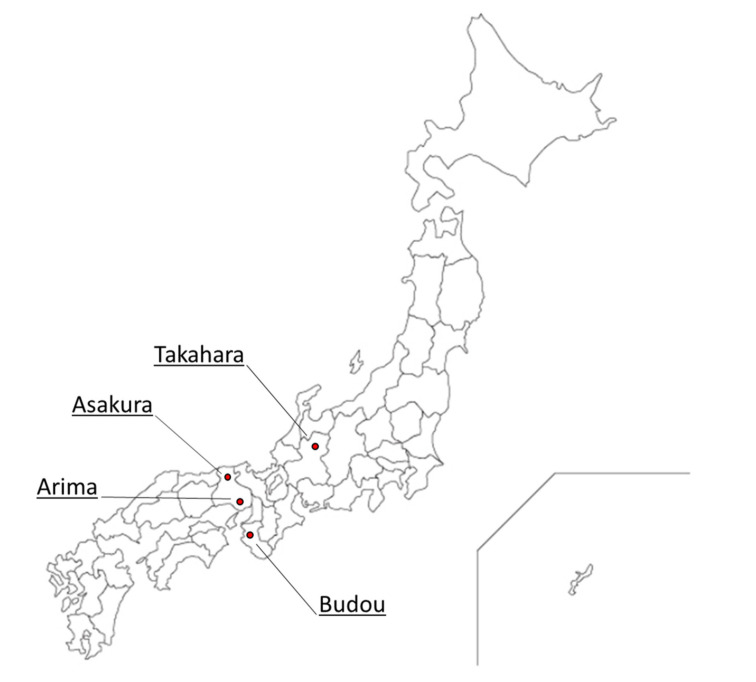
Geographical origins of representative lineages of Japanese pepper in Japan.

In order to expand the use of Japanese pepper for its health benefits, it is important to understand the characteristics of the various lineages. There are several lineages of pepper plants in Japan. Various lineages of Japanese pepper are grown in each region of Japan; Asakura, Arima, Budou, and Takahara are representative lineages (Figure 1). Although there are several such lineages, it was not known whether each of these lineages is genetically distinct from the others. Therefore, our research group has previously conducted a genome-level classification of cultivated and wild species of Japanese pepper in various regions of Japan using RAD-Seq (Restriction Site Associated DNA Sequencing) [21] and has found that each lineage can be identified at the DNA level. The next challenge is to analyze and identify the differences in the properties of these different lineages. However, there were limitations in the identification of lineages based on morphological characteristics, such as the productivity and the presence or absence of spines. For example, spineless Japanese pepper is considered monophyletic, and its subspecies name is *Z. piperitum* (L.) DC forma *inerme* (Makino) Makino. However, our DNA analysis revealed that spineless Japanese peppers were not monophyletic. Although the characteristics of the volatile compounds of each lineage of Japanese pepper fruits and their changes over time have been studied [1,3,4], no studies have focused on the characterization, identification, and classification of each lineage by analyzing their volatile compounds. Furthermore, no investigations have been conducted as to whether the differences in volatile compounds between lineages are responsible for the differences in flavor perception in humans. This information can be useful in many ways, such as identifying lineage, proposing utilization methods based on the characteristics of each lineage, and educating, transmitting, protecting, and developing food culture. In this study, we conducted gas chromatography (GC) and GC/mass spectrometry (GC/MS) analyses of volatile compounds. We performed a sensory evaluation of flavor by an analytical panel using commercially available dry powdered Japanese peppers from different regions and classified and compared the results.

## 2. Results

### 2.1. Analysis of Volatile Compounds

**Figure 2:**
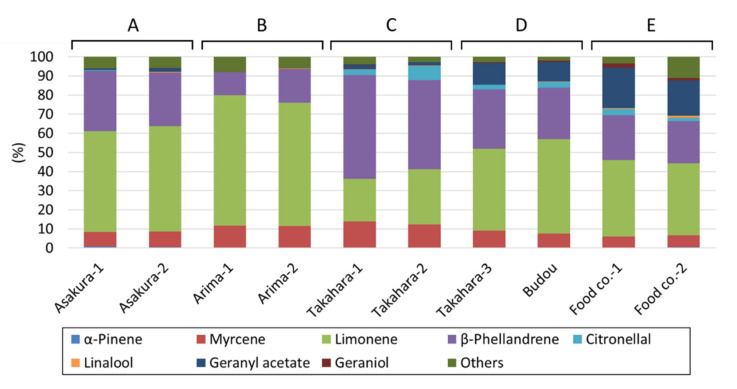
Volatile compounds of each Japanese pepper product. Data represent mean (peak area (%), *n* = 3 for each sample). “Food co.” means a product from a food company.

**Figure 3:**
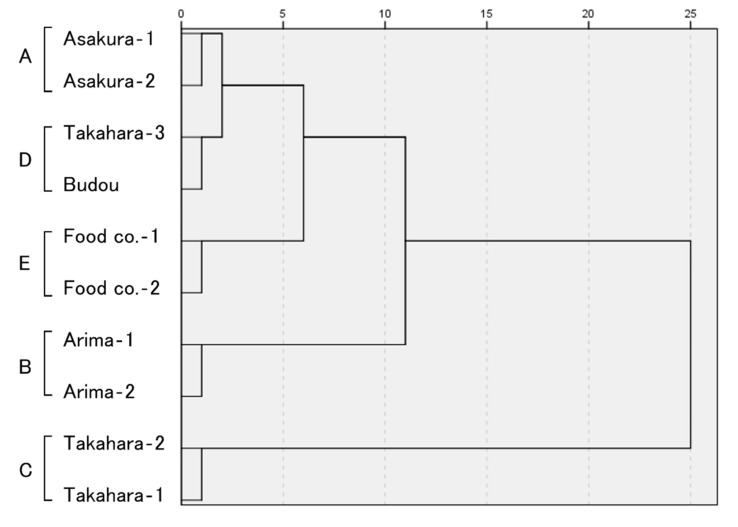
Dendrograms based on the volatile compounds of Japanese pepper.

The results of the GC and GC/MS analyses are shown in Figure 2 and Table S1, and the cluster analysis of these results is shown in Figure 3. Limonene, β-phellandrene, citronellal, and geranyl acetate were detected as the major volatile compounds of Japanese peppers. Each sample was classified into the following five groups as a result of cluster analysis based on the relative peak areas of each component. In group A (Asakura lineage group), limonene was the dominant component, followed by β-phellandrene. Little citronellal or geranyl acetate was detected. In group B (Arima lineage group), limonene was the dominant component, followed by β-phellandrene. Little citronellal or geranyl acetate was detected. The percentage of β-phellandrene was lower in group B than in group A (limonene; group A: 52.6 ± 0.8 and 55.1 ± 1.1%; group B: 68.4 ± 2.7 and 64.7 ± 2.4%, β-phellandrene; group A: 31.5 ± 0.3 and 28.0 ± 0.4%; group B: 12.0 ± 0.6 and 17.6 ± 0.1%). In group C (Takahara lineage group), β-phellandrene was the dominant component, followed by limonene. Citronellal and geranyl acetate were also detected (limonene; 22.4 ± 0.2 and 29.0 ± 0.5%, β-phellandrene; 54.2 ± 0.6 and 46.6 ± 0.8%). In group D (Takahara/Budou lineage groups), limonene was the dominant component, followed by β-phellandrene. High proportions of citronellal and geranyl acetate were detected (geranyl acetate; 11.2 ± 0.2 and 10.2 ± 0.1%). In group E (widely used commercially available products), limonene was abundant, with β-phellandrene and geranyl acetate present in nearly equal amounts (β-phellandrene; 23.5 ± 0.4 and 22.0 ± 0.3%, geranyl acetate; 21.3 ± 0.3 and 18.4 ± 0.3%). Other peaks were also abundant.

### 2.2. Sensory Evaluation

**Figure 4:**
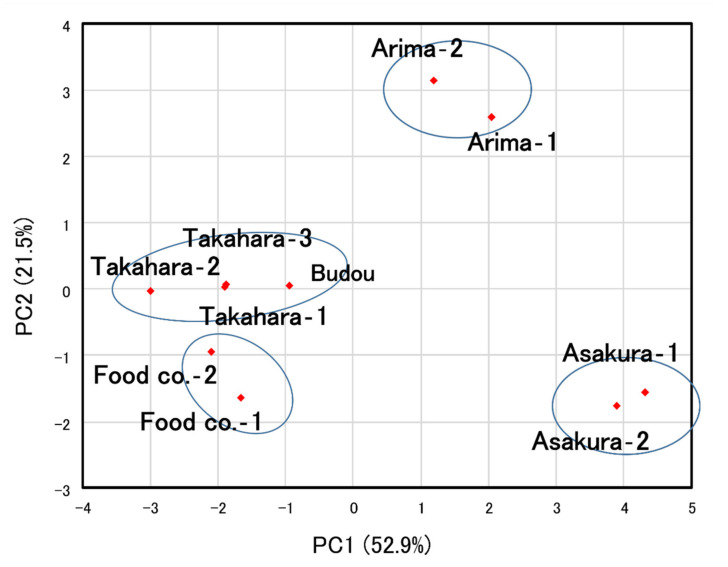
Classification of Japanese pepper based on sensory evaluation. The contribution rate of each principal component is shown in parentheses.

**Figure 5:**
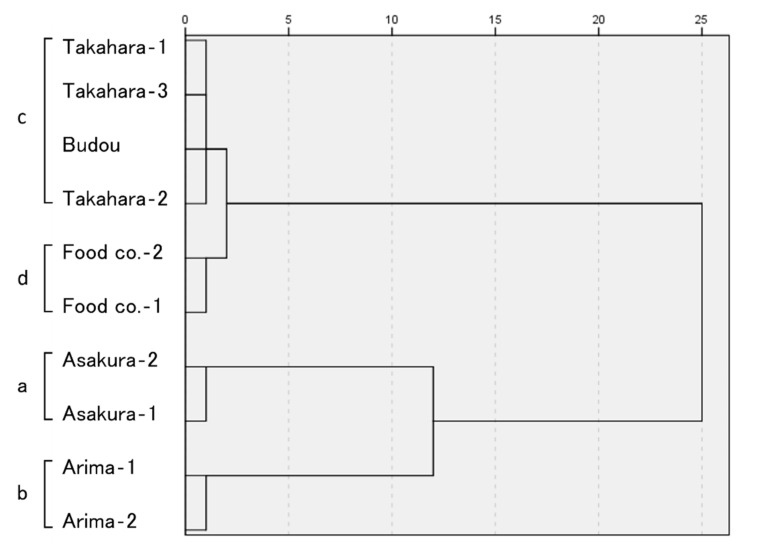
Dendrograms based on sensory evaluation of Japanese pepper.

Figure 4 and Figure 5 and Table S2 show the results of the sensory evaluation. The ten samples were divided into four well-separated groups by cluster analysis. These were the Asakura lineage group, the Arima lineage group, the Takahara and Budou lineage group, and the group of widely used commercially available products.

The classification of groups A, B, and E by volatile compound analysis was consistent with the classification based on sensory evaluation. Groups C and D were classified as a single group (Table 1).

## 3. Discussion

In this study, we conducted GC and GC/MS analyses of volatile compounds and a sensory evaluation of flavor by an analytical panel using various commercial dry powdered Japanese peppers from different regions, classified and compared the results. GC and GC/MS analysis was classified into five groups, and sensory evaluation was classified into four groups, and the results of both classifications were in good agreement.

As in previous reports, the GC and GC/MS analysis detected limonene, β-phellandrene, citronellal, and geranyl acetate as the characteristic components of Japanese pepper [1,2,3]. In this study, limonene and β-phellandrene were the dominant components in all the groups. Cluster analysis based on GC and GC/MS analysis showed that the Japanese pepper samples from various regions were classified into two major clusters: the Asakura, Arima, Takahara/Budou lineages, and commercially widely available product groups in one and the Takahara lineage in the second group. These are groups (A, B, D, and E) and the Takahara lineage (C). This classification may be due to the former group (A, B, D, and E) being limonene-dominant, whereas the latter (C) was β-phellandrene-dominant. Among the limonene-dominant groups (A, B, D, and E), the Arima lineage group (B) had a particularly high limonene content, which may contribute to the formation of a second distinct cluster. The next most significant contributor was the ratio of citronellal and geranyl acetate. Groups A and D were the most similar in the cluster analysis. However, one-way analysis of variance showed significant changes in the relative peak areas of citronellal and geranyl acetate (*F* = 2737.853, *P* < 0.0001) among the four samples of group A and D, and the significant differences were found especially between the respective samples of groups A and D (*P* < 0.0001). Thus, the abundances of citronellal and geranyl acetate differed significantly between the two groups. In recent years, as in this study, GC and GC/MS analysis of the gas-phase portion of vials containing samples (headspace method) has been reported to identify propolis from different origins and characterize *Astragalus* species [22,23]. These results indicate that the ratios of limonene, β-phellandrene, citronellal, and geranyl acetate can serve as discriminating elements, suggesting that GC and GC/MS analysis may be useful not only in assessing the quality and characteristics of Japanese pepper but also in distinguishing between lineages.

On the other hand, the classification based on sensory evaluation first divided the peppers into two groups: the Asakura and Arima lineage groups (a and b) and the Takahara-, Budou lineages, and the commercially available products group (c and d). The first group was then sub-divided into Asakura (a) and Arima lineages (b), while the other group was divided between the Takahara and Budou lineages (c) and the commercially widely available products group (d). Considering the results of GC and GC/MS analysis, the initial classification into two main groups may be due to the ratio of citronellal and geranyl acetate present. Limonene, citronellal, and geranyl acetate have characteristic flavors. In several reports, the aromatic description of limonene is expressed as “lemon”, “orange peel”, and “sweet” [24,25,26]. In other words, in the Asakura and Arima lineages (a and b), which were dominated by limonene with little to no citronellal or geranyl acetate detected by GC and GC/MS analysis, the flavor derived from limonene predominated. By contrast, in several reports, the aromatic description of citronellal and geranyl acetate are expressed as “citrus”, “strong”, “warm”, “floral”, and “geranium-like” [24,25,26]. The Takahara and Budou lineages and commercially available product groups (c and d) have volatile compounds that are qualitatively different from limonene. This difference may have contributed to the sensory evaluation results. In the sensory evaluation, the Asakura lineage group and the Arima lineage group had quite distinctive flavors compared to the others. Therefore, PCA placed these two groups more distant from the rest of the groups. As a result, the remaining two groups were positioned close together. Although cluster analysis divided the remaining groups into two, they were very close to each other. Hence, the results of PCA are consistent with those of cluster analysis. Jiang et al. evaluated the Budou lineage using the aroma extract dilution analysis (AEDA) method, in which each volatile compound separated by GC is sniffed by the human nose and reported that citronellal is the major contributor to the flavor of Japanese pepper [1]. In addition, GC and GC/MS analysis of Andaliman pepper (*Zanthoxylum acanthopodium* DC), a plant in the same genus as the Japanese pepper, detected myrcene, limonene, citronellal, and geranyl acetate, as in this study; it has been reported that citronellal is the highest flavor contributor [26]. It should be noted, however, that specific volatile compounds are known to be pleasant at low concentrations but may be unpleasant at high concentrations. Indeed, it has been reported that the subjective evaluation by humans changes with temperature and concentration in the essential oil of lemon, which belongs to the same Rutaceae family as Japanese pepper [27]. Thus, although citronellal and geranyl acetate have previously been suggested to have significant sensory importance due to their flavor quality and intensity, this study showed that ratios in which these occur are also very important in determining a human’s impression of the flavor of Japanese pepper.

On the other hand, the results of multiple factor analysis and cluster analysis of sensory evaluation showed clearly separated Asakura- (a) and Arima lineages (b) within the Asakura- and Arima lineage groups (a and b). This is in contrast to the relative similarity between the Takahara and Budou lineage groups (c) and the commercially available products group (d) within the group of Takahara-, Budou lineages, and the commercially available products group (c and d). GC analysis of tomato leaves has shown that the detection thresholds for limonene and β-phellandrene in water are 10 and 500 ppb, respectively [28], indicating that humans are more sensitive to limonene scents than β-phellandrene. There was a difference in the relative amount of limonene between the Asakura and Arima lineages, which may have contributed to the classification results of the sensory evaluation. Linalool, geraniol, and myrcene are also known to have low thresholds and be of high sensory importance [1]. Although the relative amounts were small, there was a slight difference between the Asakura and Arima lineages; the possibility that these components contributed to the classification results cannot be excluded.

The GC and GC/MS analysis showed that limonene was the most abundant component in the Takahara and Budou lineage group (D), whereas β-phellandrene was the most abundant in the Takahara lineage group (C). Although they seemed to be very distinctive on the basis of GC and GC/MS analysis, they were placed in the same cluster based on sensory evaluation (c). These results suggest that the contribution of β-phellandrene to the sensory evaluation is small and that the ratios of limonene, citronellal, and geranyl acetate present are more important. Thus, although β-phellandrene is very useful for identifying lineages, it does not contribute to identification by sensory evaluation; therefore, its contribution to consumer preferences may be low.

The reason this study was performed using the headspace method, is that it is possible that higher-boiling components were not adequately analyzed. However, this method has the advantage of being more convenient than other analytical methods, such as organic solvent extraction, steam distillation, or vacuum distillation. Although the sample size was small, it reflected the characteristics of each group; thus, the applicability and feasibility of using this method for lineage identification are high. This study also demonstrated that differences in the composition of volatile compounds of Japanese peppers are responsible for the differences in fragrances perceived by humans. The important point is not superiority or inferiority but to use them according to their respective characteristics. Our results provide an important basis for proposing cooking and utilization methods that take advantage of the unique characteristics of each lineage based on scientific evidence. Using these findings and classification at the DNA level, it is important to protect and transmit the disappearing local food ingredients and food culture in various regions through education, branding, and regional revitalization.

## 4. Materials and Methods

### 4.1. Materials

Ten dry powdered *Zanthoxylum piperitum* products from the different lineages used in this study are shown in Table 1. Three kinds of products using Asakura- and Arima lineages (No. 2, 3, 4) were generously provided by Yabu Partners Co., Ltd. (Hyogo, Japan) and Arima GOSHOBO Co., Ltd. (Hyogo, Japan), respectively. Seven types of products (No. 1, 5–10) were purchased from a commercial market in Japan.

Authentic standards (purity ≥95 %) of α-pinene, myrcene, limonene, linalool, geranyl acetate, and geraniol were purchased from FUJIFILM Wako Pure Chemical Co. Ltd. (Osaka, Japan). Citronellal was purchased from Sigma-Aldrich (St. Louis, MO, USA). The MonoTrap^TM^ sampling kit was purchased from GL Science (Shinjuku, Tokyo, Japan).

### 4.2. Extraction of Volatile Compounds

The volatile compounds of Japanese pepper were extracted using the monolithic material sorptive extraction (MMSE) method [29]. Briefly, the MonoTrap RCC18 was placed at a fixed position in the headspace of the vial containing 2.0 g of a sample. After incubation for 90 min at 60 °C, the volatile compounds absorbed in the MonoTrap RCC18 were extracted by ultrasonication for 5 min with 200 μL of dichloromethane. The extracted solution was directly injected into the GC and GC/MS to analyze the volatile compounds.

### 4.3. Analysis of Volatile Compounds

The volatile compounds were identified using GC and GC/MS. Their data were compared with those of authentic standards and previous reports. However, the presence of β-phellandrene was identified by comparing its retention time and ion peaks to previous reports due to the unavailability of an authentic standard [1,23,28].

The GC analysis was carried out on a Shimadzu GC-17A system equipped with an AOC-20i auto-sampler, a flame ionization detector (FID), and an InertCap^TM^ Pure-WAX (30 m length × 0.25 mm I.D. × 0.25 µm film thickness) column. Helium was used as a carrier gas at a constant pressure of 50 kPa. The injector and FID temperatures were maintained at 240 °C and 250 °C, respectively. The injected sample volume was 2.0 μL, and a split injection with a ratio of 1:100 was used. The oven temperature was programmed to increase from 40 to 240 °C at 3 °C/min, with initial and final hold times of 5 and 10 min, respectively. A blank run performed under the same conditions as the actual analysis confirmed the absence of contamination peaks.

Mass spectrometry was conducted using a GC/MS JEOL JMS-Q1000GC system equipped with a DB-WAX (30 m length × 0.25 mm I.D. × 0.25 µm film thickness) column. Analysis was performed under the same conditions as those used to analyze volatile compounds. The MS was measured in the EI mode.

### 4.4. Sensory Evaluation

Sensory evaluations were performed by 10 analytical panelists selected from neighbors of the Institute of Food Research, National Agriculture and Food Research Organization (NARO), according to international standards (ISO 8586). The panel consisted of nine females and nine males aged 39–59 years (48 ± 5 years). They had participated in the sensory evaluation of various products for several years, but they did not have experience with Japanese pepper.

Napping [30,31,32] was performed to determine the similarities among samples according to their sensory characteristics. Then, 4 g of each sample was placed in a screw-top amber glass vial (φ35 mm × 78 mm), and a 3-digit random code was attached. Each panelist was presented with the samples simultaneously and randomly. Each panelist sniffed the headspace of the sample vial and then placed the vial on a white A3 sheet of paper based on the similarities or dissimilarities between the samples to create each panelist’s perceptual map of samples.

The sensory evaluation was performed in an isolated booth at a room temperature of 23 ± 2 °C. The Institutional Review Board of the Food Research Institute of NARO (28NFRI-0001) approved the use of human subjects in this research.

### 4.5. Data Analysis

For the GC analysis, the relative peak area values of each volatile compound were used as variables for cluster analysis using the Ward method with SPSS Statistics (version 26; IBM, Armonk, NY, USA). For the sensory data, the bottom and left corners of each sheet were set as the origin, and the coordinates of each product were collected in centimeters. The data were compiled in a product-by-assessor table. The matrix was then subjected to multiple factor analysis using FIZZ Calculations (version 2.61; Biosystemes, Dijon, France). The resultant coordinates obtained via the multivariate analyses of each sample were then subjected to cluster analysis using the Ward method. Cluster analysis was performed using SPSS Statistics (version 25; IBM, Armonk, NY, USA).

## Figures and Tables

**Table 1:** Japanese pepper products used in the study.

No.	Product	GC and GC/MS Analysis	Sensory Evaluation
1	Asakura-1	A	a
2	Asakura-2	A	a
3	Arima-1	B	b
4	Arima-2	B	b
5	Takahara-1	C	c
6	Takahara-2	C	c
7	Takahara-3	D	c
8	Budou	D	c
9	Major food manufacturing company in Japan-1 (Food co.-1)	E	d
10	Major food manufacturing company in Japan-2 (Food co.-2)	E	d

## Data Availability

The data underlying this article will be shared on reasonable request to the corresponding author.

## Supplementary Materials

The following supporting information can be downloaded at: https://www.mdpi.com/article/10.3390/molecules27154946/s1, Table S1: Relative value of volatile compounds of each Japanese pepper product; Table S2: Sensory coordinates of each Japanese pepper product.

## Sample Availability

Samples of the compounds are available from the authors.
