# Supplementary material for: Classification of Japanese Pepper (Zanthoxylum piperitum DC.) from Different Growing Regions Based on Analysis of Volatile Compounds and Sensory Evaluation
Source: Molecules. 2022 Aug 3;27(15):4946. doi: 10.3390/molecules27154946 (PMC9370631; doi:10.3390/molecules27154946)
Supplement: Supplementary file 1 [file molecules-27-04946-s001.zip › molecules-1804368-supplementary.pptx]

## Slide 1
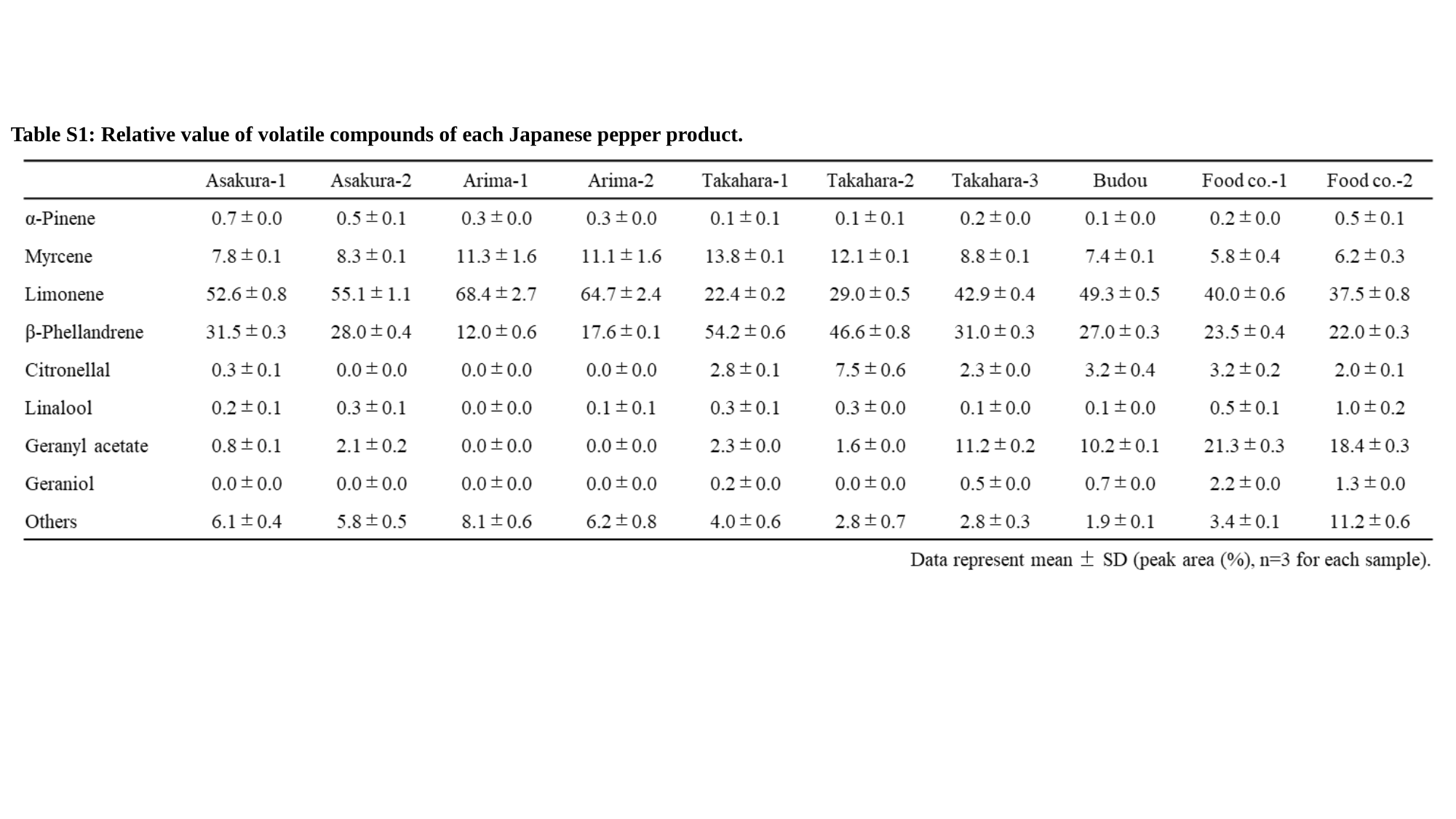

Table S1: Relative value of volatile compounds of each Japanese pepper product.

## Slide 2
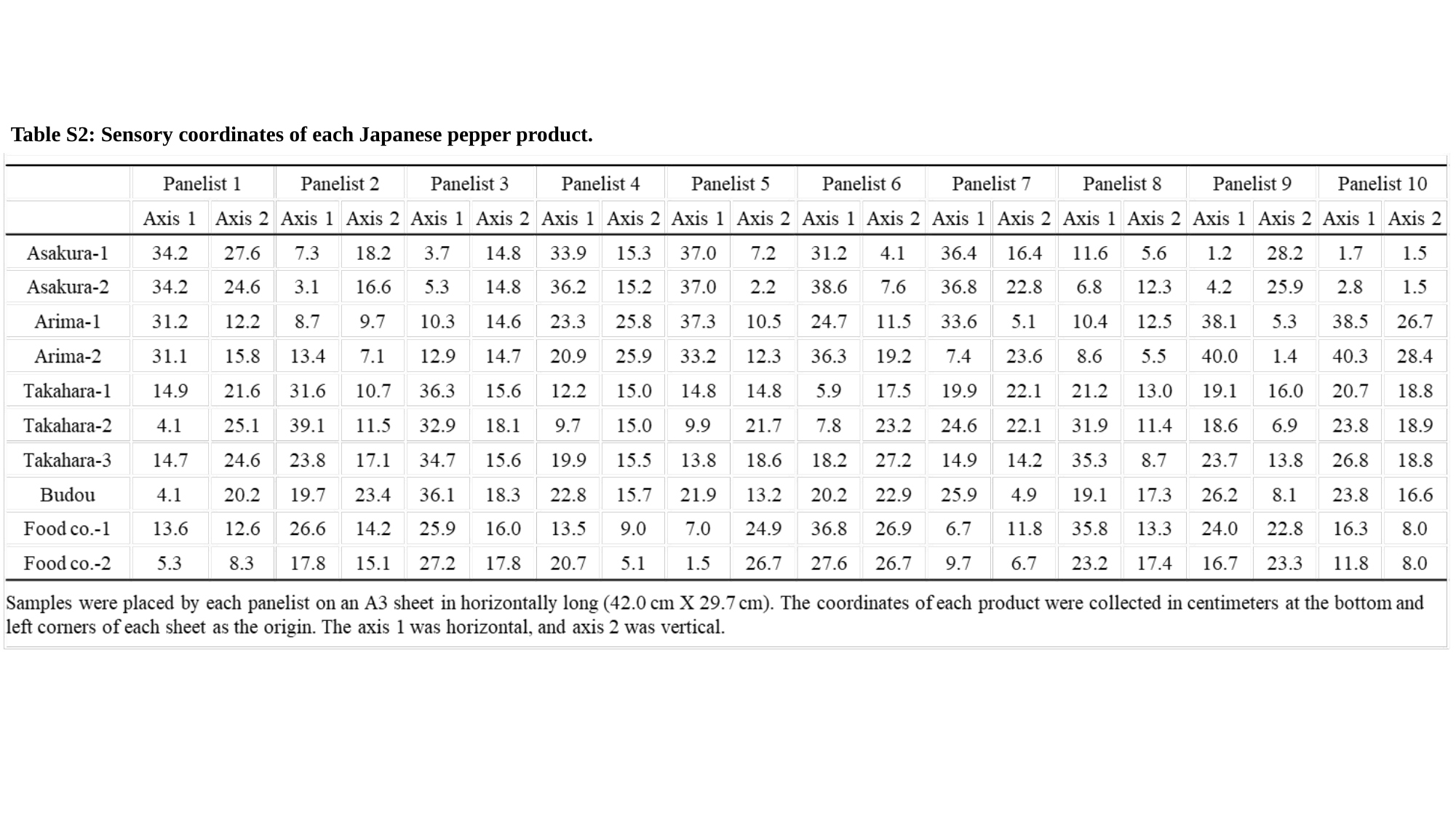

Table S2: Sensory coordinates of each Japanese pepper product.
